# Avascular necrosis in proximal humeral fractures in patients treated with operative fixation: a meta-analysis

**DOI:** 10.1186/1749-799X-9-31

**Published:** 2014-04-27

**Authors:** Jiaming Xu, Changqing Zhang, Tao Wang

**Affiliations:** 1 Department of Orthopedics, Shanghai Jiaotong University Affiliated Sixth People's Hospital, 600 Yishan Road, Shanghai 200233, China

**Keywords:** Avascular necrosis, Operative fixation, Conservative treatment, Proximal humeral fracture, Meta-analysis

## Abstract

**Background:**

Proximal humeral fractures are common lesions of the elderly, but there are no established treatment guidelines. A surgical treatment for comminuted and displaced fractures of the proximal humerus was developed and is still evolving. The aim of this study was to perform a quantitative review to evaluate the risk of avascular necrosis (AVN) in patients with proximal humeral fractures who were treated by operative fixation compared with conservative treatment.

**Methods:**

We searched the PubMed, MEDLINE, Springer, Elsevier Science Direct, Cochrane Library, Google Scholar, China National Knowledge Infrastructure (in Chinese), and Wanfang database (in Chinese) up to December 2013 to identify studies related to operative fixation and AVN in patients with proximal humeral fractures.

**Results:**

Seven studies with a total of 291 patients (142 operative fixation cases and 149 conservative treatment cases) with proximal humeral fractures were considered in the meta-analysis. The overall meta-analysis showed no significant difference in the incidence of AVN between the two groups [odds ratio (OR) 1.42, 95% confidence interval (CI) 0.33–6.11, *p* = 0.64]. The subgroup meta-analysis by study design (retrospective/prospective), sample size (≤40/>40), and ethnicity (European/Asian) demonstrated similar results. However, the subgroup analysis by specific operative approach (plate fixation/tension band wiring fixation/others) indicated that plate fixation was associated with a higher rate of AVN than conservative treatment (OR 0.20, 95% CI 0.05–0.76, *p* = 0.019).

**Conclusions:**

Plate fixation was associated with a higher risk of AVN development than conservative treatment in patients with proximal humeral fractures.

## Background

Although proximal humeral fractures are common lesions of the elderly, accounting for 5% of all fractures and 45% of all humeral fractures [1-3], there is no consensus regarding their treatment. The majority of patients suffering this injury have nondisplaced or minimally displaced two- or three-part fractures. They are treated conservatively with a modified Velpeau bandage or in a sling, both of which require immobilization for more than 2 weeks [4,5]. This conservative treatment of severe displacement of proximal humeral fracture fragments, however, often yields poor functional results [6]. Therefore, operative treatment of comminuted and displaced fractures of the proximal humerus was developed and has been evolving in recent years [7].

Currently, many surgical solutions such as operative fixations (retrograde percutaneous pin fixation [5], transcutaneous fixation with Kirschner wires [8], and tension band osteosynthesis [9]) and hemiarthroplasty [10] are applied to treat these lesions, ranging from percutaneous pinning to shoulder arthroplasty [1]. These surgical solutions have added to the surgeon's armamentarium of methods to treat proximal humeral fractures and have been suitable for patients with various conditions. Surgical solutions, however, do little to limit the activities of patients compared with the conservative approach. Also, open operative techniques pose a higher risk of the patient developing avascular necrosis (AVN) of the humeral head [6].

Whether the incidence of AVN is less after operative fixation versus conservative treatment for proximal humeral fractures is controversial. In a multicenter analysis reported by Schai et al., open reduction and internal fixation was associated with significantly less AVN than conservative treatment of humeral head fractures [11]. In contrast, Fjalestad and coworkers reported that surgical treatment of displaced proximal humeral fractures was associated with obviously more AVN development than conservative treatment [5].

There was thus a need to gather the current best evidence of AVN incidence in patients with proximal humeral fractures in regard to operative fixation versus conservative treatment. Therefore, we conducted a systematic review of published research and applied a meta-analysis to integrate the results quantitatively.

## Methods

### Source of material

We used established search strategies and retrieved literature in a systematic way from various databases, including PubMed, MEDLINE, Springer, Elsevier Science Direct, Cochrane Library, Google Scholar, CNKI (China National Knowledge Infrastructure, in Chinese), and Wanfang (in Chinese) up to December 2013. The key words ‘avascular necrosis’, ‘operative fixation’, and ‘proximal humeral fractures’ were used for the searches. References from retrieved papers were checked for additional studies. We collected data only from fully published papers—not from meeting or conference abstracts. The publication date was not restricted in our research.

### Inclusion and exclusion criteria

Studies were included in the meta-analysis if they met the following criteria. The study had to contain (1) the occurrence rates of AVN in patients with proximal humeral fractures (prospective, retrospective, and cross-sectional studies), (2) operative fixation versus conservative treatment data, and (3) the effect size reported as odds ratios (ORs) and 95% confidence intervals (CIs) or it could be calculated. The sample size and age range were not limited. Studies were excluded if the study (1) described only conservative treatment data in a review or report, (2) was a reduplicated study, or (3) did not compare operative fixation with conservative treatment.

### Data extraction and quality evaluation

Articles were reviewed and filtered out independently by two investigators according to our criteria. The data were then extracted independently in duplicate using a standardized form to assess eligibility for inclusion. Data items included study details (e.g., the first author's name, research year(s) of the study, year of the study's publication, location of participants, design of the study, follow-up time) and characteristics of the participants (e.g., age, sex, sample size). Discrepancies were resolved by consensus.

Evaluation of quality mainly included the sample size and recruitment of the studies. The papers were first selected by reading the document titles and abstracts. Then, we read the full text of each paper to determine whether the study conformed to our inclusion criteria. Two investigators independently completed this task. Assuming that differences would occur, agreements were to be reached by discussion.

### Meta-analysis methods

The meta-analysis was performed in fixed or random effect models as appropriate. The effect sizes of the ORs with 95% CIs were pooled to assess the AVN incidence associated with operative fixation in proximal humeral fractures compared with conservative treatment. Heterogeneity among studies was evaluated by Cochran's *Q*-statistic [12] and *I*^2^ parameter testing [13]. Also, *p* < 0.05 or *I*^2^ > 50% was considered to indicate a heterogeneous nature. When substantial heterogeneity was detected, we calculated the summary ORs and their 95% CIs with the DerSimonian and Laird method in the random effect model [14]. If heterogeneity was not detected, the pooled estimate was presented based on the Mantel-Haenszel method in the fixed effect model [15]. To test the reliability of the results, we undertook a sensitivity analysis by repeating the meta-analysis after removing one study each time.

We further conducted subgroup analysis according to the study design (retrospective/prospective), sample size (≤40/>40), ethnicity (European/Asian), and specific operative approach (plate fixation/tension band wiring fixation/others) to investigate the impact of study characteristics on our outcomes. Publication bias was also assessed by Egger's regression asymmetry test [16].

Analyses were performed using the software Review Manager 5.1 (Cochrane Collaboration: http://ims.cochrane.org/revman) and the STATA software package version 11.0 (Stata, College Station, TX, USA). All *p* values were two-sided. A value of *p* < 0.05 was considered to indicate statistical significance.

## Results

### Characteristics of eligible studies

**Figure 1:**
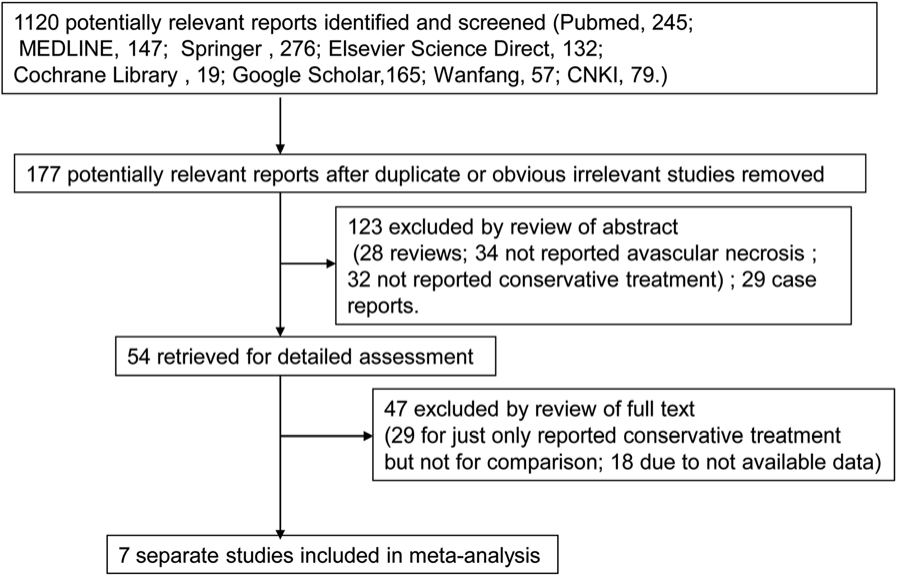
Flow diagram of the study selection process.

The details of the literature search are presented in a flow diagram (Figure 1). We identified 1,120 papers potentially relevant to the search terms (PubMed, 245; MEDLINE, 147; Springer, 276; Elsevier Science Direct, 132; Cochrane Library, 19; Google Scholar, 165; Wanfang, 57; CNKI, 79). There were 117 studies after removing duplicates. During the screening phase based on titles and abstracts, 123 articles were excluded (28 were review articles; 34 did not report AVN; 32 did not report conservative treatment; 29 were case reports). Among the remaining 54 studies for full publication review, only 7 met the inclusion criteria after full publication review.

The characteristics of the seven included studies [5,9,11,17-20] are presented in Table 1. The studies were published between 1988 and 2012. A total of 291 patients (142 operative fixation cases and 149 conservatively treated cases) with proximal humeral fractures were considered in this meta-analysis. The studies' sample sizes ranged between 12 and 93, and patients' ages ranged between 65.2 and 75.0 years. Various operative approaches were conducted. Plate fixation was performed in two of the seven studies [11,20], tension band wiring fixation in two studies [9,18], and Steinmann pin fixation in one study [17]. Also, AO angular blade plate, screw fixation and cerclage, antegrade screw nails, and retrograde pin fixation were used in one study each [5]. Data for single operative approaches were not provided. In the remaining article [19], no details were given for the open reduction with internal fixation approach.

**Table 1:** Characteristics of studies included in the meta-analysis

**Study**	**Country**	**Ethnicity**	**Sample size**	**Study design**	**Operative approaches**	**Average age, years**
Fan Y, 2012 [20]	China	Asian	35	Retrospective	Open reduction and locking plate internal fixation	75.0
Fjalestad T, 2005 [5]	Norway	European	51	Retrospective and prospective	AO angular blade plate, screw fixation and cerclage, antegrade screw nails, or retrograde pin fixation	75.0
Ilchmann T, 1998 [9]	Sweden	European	24	Retrospective	Tension band wiring fixation	65.2
Kristiansen B, 1988 [17]	Denmark	European	24	Prospective	Steinmann pin	69.0
Schai P, 1995 [11]	Switzerland	European	93	Retrospective	Minimal internal fixation or plate fixation	67.5
Zyto K, 1995 [19]	Sweden	European	35	Retrospective	Open reduction and internal fixation, not in detail	71.0
Zyto K, 1997 [18]	Sweden	European	29	Prospective	Tension band wiring fixation	74.0

### Overall analysis

**Figure 2:**
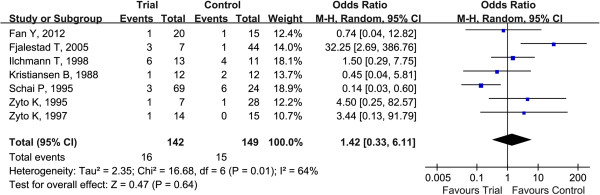
**Forest plots for ORs for AVN in proximal humeral fractures associated with operative fixation versus conservative treatment.***Squares* represent the effect size for the odds ratios of AVN in proximal humeral fractures associated with operative fixation versus conservative treatment. The sizes of the *squares* are proportional to the sizes of the cohorts. *Error bars* represent 95% confidence intervals (*CIs*). *Diamond* represents the pooled estimates within each analysis.

In an overall analysis of the seven selected studies, the heterogeneity test showed that there were heterogeneities (*Q*^2^ = 16.68, *I*^2^ = 64%, *p* = 0.01) among studies. Thus, the random effect model was applied. The results showed that there was a relatively higher risk of AVN in patients with proximal humeral fractures who underwent operative fixation than in those treated conservatively (Figure 2) (total OR = 1.42, 95% CI 0.33–6.11, *p* > 0.05), but the difference was not statistically significant. The result of Egger's linear regression test (Table 2) indicated that there was no publication bias in this study (*t* = 1.52, *p* > 0.05).

**Table 2:** Pooled odds ratio for operative fixation versus conservative treatment in the meta-analysis

**Subgroups**	**Sample size**		**Number of studies**	**Random model**			**Test of heterogeneity**			**Egger's test for publication bias**	
	**Case**	**Control**		**OR (95% CI)**	***Z***	***p *** **value**	***Q***	***p *** **value**	***I***^**2 **^**(%)**	***t***	***p *** **value**
Overall effects	142	149	7	1.42 (0.33–6.11)	0.47	0.64	16.68	0.01	64	1.52	0.19
Study design											
Retrospective	109	78	4	0.73 (0.15–3.55)	1.21	0.23	6.89	0.08	56	0.99	0.43
Prospective	26	27	2	1.03 (0.17–6.40)	0.03	0.98	0.92	0.34	0.0	-0.03	0.98
Sample size											
Sample size ≤40	66	81	5	1.36 (0.48–3.86)	0.60	0.55	1.86	0.76	0.0	0.30	0.79
Sample size >40	102	87	3	1.52 (0.10–24.11)	0.29	0.77	14.35	0.001	86.1	-	-
Ethnicity											
European	122	134	6	1.60 (0.30–8.51)	0.55	0.58	16.64	0.005	70	1.62	0.18
Asian	20	15	1	0.74 (0.04–12.82)	0.21	0.83	-	-	-	-	-
Operative approaches											
Plate fixation	89	39	2	0.20 (0.05–0.76)	2.35	0.019	1.01	0.315	0.8	-	-
Tension band wiring fixation	27	26	2	1.77 (0.41–7.70)	0.76	0.445	0.20	0.657	0.0	-	-
Others	26	84	3	4.01 (0.31–45.26)	1.07	0.285	5.68	0.058	64.8	0.03	0.983

### Subgroup analysis

The outcomes of the subgroup analysis stratified by study design, sample size, ethnicity, and operative approach are shown in Table 2. The results indicated that a nonsignificant increase of AVN in patients with proximal humeral fractures was consistent in the subgroup analyses when stratified by sample size. In the study design analysis, patients in retrospective studies showed that operative fixation had a nonsignificantly decreased risk of AVN (OR 0.73, 95% CI 0.15–3.55, *p* > 0.05). In the stratified analysis by ethnicity, Asian patients (OR 0.74, 95% CI 0.04–12.82) showed less risk of developing AVN than European patients (OR 1.60, 95% CI 0.30–8.51) when treated with operative fixation. The result of the subgroup analysis by operative approach demonstrated that plate fixation had a significantly increased risk of AVN compared with conservative treatment (OR 0.20, 95% CI 0.05–0.76, *p* = 0.019), whereas tension band wiring fixation showed a risk similar to that with conservative treatment (OR 1.77, 95% CI 0.41–7.70, *p* > 0.05).

### Sensitivity analysis and publication bias

We performed the sensitivity analysis by removing one study each time and rerunning the model to determine the effect on each overall estimate. By omitting the Fjalestad et al. study [5], for instance, the pooled OR was changed to 0.64 (95% CI 0.29–1.44, *p* > 0.05), and the heterogeneity test showed that there were heterogeneities (*I*^2^ = 38%, *p* = 0.15). Also, the estimates changed little, which implied that our results were statistically reliable.

Egger's linear regression test was performed to assess the publication bias of the articles. In this study, the total *p* value for AVN was 0.19, which indicated no statistical significance for publication bias.

## Discussion

Previous studies [5,9,11,18,21] have reported the risk of AVN after operative fixation versus conservative treatment in patients with proximal humeral fractures. These studies showed mixed results because of their small sample sizes or low statistical power. In the present meta-analysis, we combined and reanalyzed seven studies that contained 291 patients (142 operative fixation cases and 149 conservatively treated cases) achieve an integrative knowledge of AVN risk with operative fixation.

Findings from the meta-analysis showed that in patients with proximal humeral fractures, operative fixation was associated with a nonsignificantly higher risk of AVN development than conservative treatment (OR 1.42, 95% CI 0.33–6.11, *p* > 0.05). This finding was consistent with results in a study by Ilchmann and coworkers, who found that the risk of developing AVN was at an OR (95% CI) of 1.50 (0.29–7.75) [9]. Also, the effect sizes of our meta-analysis were similar to those of Kollig and his colleagues' results (OR 1.05, 95% CI 0.14–8.02) [21]. According to the subgroup analysis, Asian patients (OR 0.74, 95% CI 0.04–12.82) and patients in retrospective studies (OR 0.73, 95% CI 0.15–3.55, *p* > 0.05) showed that operative fixation was associated with a nonsignificantly decreased risk of AVN, which was inconsistent with the overall estimates. Hence, study design and ethnicity might be factors that cause heterogeneity.

Although the overall analysis demonstrated a nonsignificant difference in the incidence of AVN after operative fixation versus conservative treatment, the subgroup analysis by operative approach showed a markedly increased risk of AVN after plate fixation compared with conservative treatment. Tension band wiring fixation and conservative treatment had similar incidences of AVN. Multiple materials have been developed for open reduction and internal fixation including cerclage wires, rotary self-locking intramedullary nails, intramedullary interlocking nails, and six-hole dynamic compression plates [22-24]. Among them, implant of a plate requires a long incision and excision of the perichondrium, causing exposure of soft tissue and even damage to the blood supply, which may contribute to the incidence of AVN.

Treatment of displaced proximal humeral fractures remains a challenge for the orthopedic surgeon [25], and there is no generally accepted strategy for treating them. Procedures using closed reduction are of course less invasive, but it is more difficult to achieve good reconstruction. On the other side, open techniques are more likely to obtain anatomical reduction and stronger fixation, but they present a higher rate of complications related to complete or partial necrosis of the humeral head, regardless of the implant used [1].

The treatment and prognosis of proximal humeral fractures have improved increasingly with the development of medical technology. The study of Poeze et al. indicated that radiographic evaluation in patients with minimally displaced proximal humeral fractures is helpful for predicting functional outcome during conservative treatment [26]. Humeral head replacement was said to be indicated for complex proximal humeral fractures with an avascular head fragment and for those with an unreconstructable fracture [27]. In addition, arthroscopy has become increasingly established in the treatment of proximal humeral fractures [28]. Therefore, the application of operative fixation in proximal humeral fractures should be further studied.

The present study was an updated meta-analysis on the risk of AVN after operative fixation of a proximal humeral fracture. There are some limitations of this study. First, only seven published studies were included in the meta-analysis. Second, significant heterogeneities were detected in the meta-analysis for clinical neurological outcome, which might be distorting the outcomes as heterogeneity is one of the major concerns in meta-analyses regarding validity [29]. Also, different ethnicities may contribute to the heterogeneity, and the patients from each country are not uniform. Therefore, the results should be interpreted with caution. Finally, the recruited studies were not all randomized controlled trials, and some of the sample sizes of the retrieved studies were small [12]. Thus, more high-quality studies are needed to obtain a more integrated, clear outcome.

## Conclusions

The findings of the present meta-analysis suggested that plate fixation in patients with proximal humeral fractures had a significantly higher risk of AVN development than conservative treatment, whereas tension band wiring fixation and conservative treatment had similar risks. Thus, further investigation with more high-quality studies should be carried out to obtain a more precise outcome.

## Competing interests

The authors declare that they have no competing interests.

## Authors' contributions

JX and CZ participated in the design of this study and performed the statistical analysis. JX carried out the study together with CZ, collected important background information, and drafted the manuscript. TW conceived of this study, participated in its design, and helped draft the manuscript. All authors read and approved the final manuscript.
